# Toxicity Evaluation and Biomarker Selection with Validated Reference Gene in Embryonic Zebrafish Exposed to Mitoxantrone

**DOI:** 10.3390/ijms19113516

**Published:** 2018-11-08

**Authors:** Lili Liu, Hua Zhu, Yanchun Yan, Peng Lv, Wei Wu

**Affiliations:** 1 Beijing Key Laboratory of Fishery Biotechnology, Beijing Fisheries Research Institute, Beijing 100068, China; lilyinmay@163.com (L.L.); zhuhua@bjfishery.com (H.Z.); 2 Graduate School, Chinese Academy of Agricultural Sciences, Beijing 100081, China; lvchosen1@163.com (P.L.); wuwei02@caas.cn (W.W.)

**Keywords:** reference genes, stability evaluation, zebrafish embryos, mitoxantrone, toxicity evaluation, biomarker selection

## Abstract

Notwithstanding the widespread use and promising clinical value of chemotherapy, the pharmacokinetics, toxicology, and mechanism of mitoxantrone remains unclear. To promote the clinical value in the treatment of human diseases and the exploration of potential subtle effects of mitoxantrone, zebrafish embryos were employed to evaluate toxicity with validated reference genes based on independent stability evaluation programs. The most stable and recommended reference gene was *gapdh*, followed by *tubα1b*, for the 48 h post fertilization (hpf) zebrafish embryo mitoxantrone test, while both *eef1a1l1* and *rpl13α* were recommended as reference genes for the 96 hpf zebrafish embryo mitoxantrone test. With *gapdh* as an internal control, we analyzed the mRNA levels of representative hepatotoxicity biomarkers, including *fabp10a*, *gclc*, *gsr*, *nqo1*, cardiotoxicity biomarker *erg*, and neurotoxicity biomarker *gfap* in the 48 hpf embryo mitoxantrone test. The mRNA levels of *gclc*, *gsr*, and *gfap* increased significantly in 10 and 50 μg/L mitoxantrone-treated 48 hpf embryos, while the transcript levels of *fabp10a* decreased in a dose-dependent manner, indicating that mitoxantrone induced hepatotoxicity and neurotoxicity. Liver hematoxylin–eosin staining and the spontaneous movement of embryos confirmed the results. Thus, the present research suggests that mitoxantrone induces toxicity during the development of the liver and nervous system in zebrafish embryos and that *fabp10a* is recommended as a potential biomarker for hepatotoxicity in zebrafish embryos. Additionally, *gapdh* is proposed as a reference gene for the 48 hpf zebrafish embryo mitoxantrone toxicity test, while *eef1a1l1* and *rpl13α* are proposed as that for the 96 hpf test.

## 1. Introduction

Zebrafish is a recent addition to vertebrate models of human disease and drug screening, rapidly contributing major insights into these fields. Nearly 70% of human genes have orthologues in zebrafish genomes [1], making zebrafish useful for assigning functions to all proteins encoded by human genes [2]. More than 75% of human genes implicated in disease have counterparts in zebrafish, providing an opportunity to analyze their roles in this model system [3]. Zebrafish tumors share conservation of expression profiles at levels different from tumors of humans [4]. The technology developments of transgenes and xenografts give rise to abundant zebrafish models of cancer, including lymphoblastic T-cell leukemia and pancreatic cancer [5]. There is some similarity between zebrafish and humans in terms of the nervous system, the cardiovascular system, and the digestive system. Zebrafish tissues and organs (brain, liver, heart, intestinal, etc.) fully develop within 72 hpf (hours post fertilization) [6]. Zebrafish embryos have been well recognized as an alternative to traditional experimental animals.

**Figure 1:**
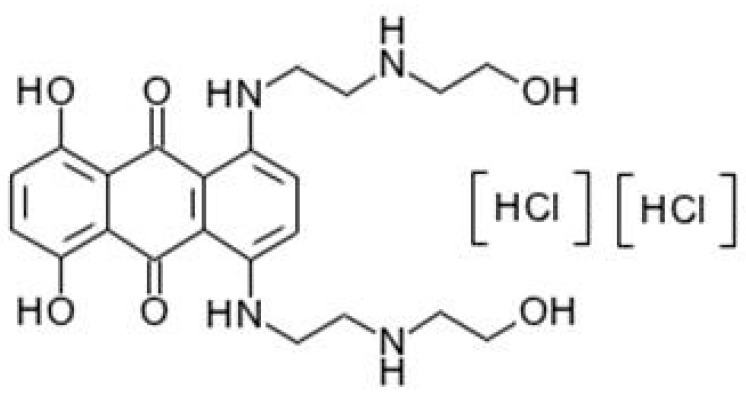
The molecular structure formula of mitoxantrone [hydorehloride].

These factors promote zebrafish as a model for research on clinical drugs. Mitoxantrone (Novantrone) is an antineoplastic well known for inhibiting DNA replication and RNA synthesis in both dividing and non-dividing cells [7]. Clinically, mitoxantrone hydrochloride (Figure 1) injection was in wide use instead of mitoxantrone without hydrochloric acid molecules. Mitoxantrone hydrochloride has the same curative effect with mitoxantrone, but with much higher solubility. Human metabolites of mitoxantrone include most unchanged protype drug and some metabolites, including mono/dicarboxylic acids [8] and cycle naphthoquinoxaline [9]. The metabolites of mitoxantrone have same bioactivity with parent structure. The bile route is the main metabolism pathway and urine route also accounts [10,11]. Notwithstanding the widespread use and promising clinical value on the chemotherapy and the knowledge attained from clinical practice, pharmacokinetics, and toxicology, the mechanism of mitoxantrone remains unclear. For example, the effect of mitoxantrone on brain tumors is limited because of its poor ability to cross the blood–brain barrier [12]. The risk and pathway of mitoxantrone-induced cardiotoxicity and heart failure are vague [13]. Cytostatic agents and most of their intermediates and metabolites are usually bioactive after in vivo metabolism, making them bioactive even in the effluent. These agents thus are toxic to aquatic organisms at very low concentrations [14,15]. As a cytostatic agent, mitoxantrone exhibits bioactivity and likely poses adverse effects to aquatic organisms. The clinical values in the treatment of human diseases and the exploration of potential subtle effects of mitoxantrone have yet to be further investigated. Zebrafish offers a new approach in the research of mitoxantrone.

Quantitative real-time PCR (qPCR), including absolute quantification and relative quantification, is a widely used method to measure transcript abundance and gene expression. The more popular relative quantification qPCR, however, bears a high possibility of inaccurate results if improper reference genes are used. Relative quantification qPCR relies on the hypothesis that reference genes are expressed at the same level under various experimental conditions in an assay. However, no internal control is constantly expressed across all developmental stages, different tissues, and experimental conditions [16]. The choice of optimal reference gene has become the most critical influence in the relative quantification qPCR method [13]. Reported reference genes in zebrafish contain *actin beta 2* (*actβ2*) [17], *glyceraldehyde-3-phosphate dehydrogenase* (*gapdh*) [18], *beta-2-microglobulin* (*β2m*) [19], *ribosomal protein L13a* (*rpl13α*) [20,21], and *eukaryotic translation elongation factor 1* (*eef1*) [22] under different experimental conditions. However, there is no validation of reference genes in zebrafish toxicity tests for mitoxantrone.

This study was designed to evaluate the toxicity of mitoxantrone on zebrafish embryos and determine reliable toxicity biomarkers. To begin with, the stability of candidate reference genes for relative quantification qPCR in zebrafish embryos exposed to mitoxantrone was evaluated with respect to different developmental stages. With the suggested internal control *gapdh*, the expression levels of a set of typical toxicity biomarker genes in 48 hpf zebrafish embryos were normalized for the developmental toxicity evaluation of mitoxantrone with respect to zebrafish. The present research suggests that mitoxantrone induces toxicity during the development of the liver and nervous system in embryonic zebrafish and that *fabp10a* might be a potential biomarker for hepatotoxicity. Additionally, *eef1a1l1* and *rpl13α* are proposed as reference genes for 96 hpf zebrafish embryo mitoxantrone tests.

## 2. Results

### 2.1. Transcript Abundance and Amplification Efficiency of Candidate Reference Genes

All 11 candidate reference genes were amplified with an amplification efficiency (E) of 90–110% and a correlation coefficient (R^2^) > 0.980 (Table 1). With diluted cDNA of embryonic zebrafish (24 and 48 hpf) from control and mitoxantrone-treated groups as templates, the qPCR assay was carried out for transcript abundance based on quantification cycle (Cq) values. Among all, *eukaryotic translation elongation factor 1 alpha 1*, *like 1* (*eef1a1la*), *polymerase (RNA) II (DNA directed) polypeptide D* (*polr2d*), *tubulin, alpha 1b* (*tubα1b*), *rpl13α*, *actβ2*, and *gapdh* were classified as median transcript abundance genes (10 < Cq < 26). The transcript level of *18S ribosomal RNA* (*18S rRNA*) was much higher than all others, with a mean Cq value of 9.44, belonging to high transcript abundance genes (Cq < 10). The last four genes *succinate dehydrogenase complex*, *subunit A*, *flavoprotein (Fp)* (*sdha*), *TATA box binding protein* (*tbp*), *hydroxymethylbilane synthase, b* (*hmbsb*), and *β2m* were low transcript abundance genes (Cq > 26).

### 2.2. Stability Evaluation of Candidate Reference Genes—NormFinder, geNorm, and BestKeeper Analysis

To evaluate the expression stability of reference genes of zebrafish embryos for mitoxantrone test in different developmental stages, three independent algorithm programs—NormFinder [23], geNorm [24], and BestKeeper [25]—were adopted to calculate the stability or variability values.

According to the NormFinder output results, the stability ranking of candidate reference genes of the 48 hpf zebrafish embryo mitoxantrone test, in ascending order of M value, was *gapdh* > *eef1a1l1* > *tubα1b* > *sdha* > *tbp* > *actβ2* > *polr2d* > *rpl13α* > *hmbsb* > *β2m* >*18S rRNA*. The higher the M value, the less stable the gene. By stepwise elimination of the gene with the highest M value (that is the least stable gene), the most stable candidate reference gene was *gapdh*. According to the geNorm output results, the stability ranking of candidate reference genes of the 48 hpf zebrafish embryo mitoxantrone test, in ascending order of M value, was *gapdh* > *sdha* > *tubα1b* > *eef1a1l1* > *actβ2* > *rpl13α* > *β2m* > *tbp* > *polr2d* > *hmbsb* > *18S rRNA*. Similar as NormFinder analysis, geNorm suggested that the most stable gene was *gapdh*. The stability was meanwhile analyzed using the BestKeeper program, with geomean values based on Cq values of candidate reference genes. Each gene was given a geomean value, and the lower ranking values represent more stable genes. The descending stability order of candidate genes in stability was *18S rRNA*> *actβ2* > *β2m* > *eef1a1l1* > *gapdh* > *tubα1b* > *sdha* > *rpl13α* > *polr2d* > *tbp* > *hmbsb* (Table 2).

As both suggested by NormFinder and geNorm programs (Table 2), *gapdh* was the most stable candidate reference gene with a minimum M value. In the second place, the *tubα1b* gene was relatively stable. The *18S rRNA* gene was not recommended as a reference gene in the present study, whereas with the least M value in BestKeeper analysis. The main reason was that the *18S rRNA* gene exhibited a very high transcript abundance. Thus, *gapdh*, followed by *tubα1b*, was recommended as a reference gene for the 48 hpf zebrafish embryo mitoxantrone test.

According to the NormFinder output results (Table 3), the stability ranking of candidate reference genes of the 96 hpf zebrafish embryo mitoxantrone test, in ascending order of M value, was *tbp* > *eef1a1l1* > *polr2d* > *actβ2* > *rpl13α* > *β2m* > *sdha* > *hmbsb* > *tubα1b* > *18S rRNA* > *gapdh*. geNorm program analyzed and ranked the gene stability as *rpl13α* > *tbp* > *actβ2* > *polr2d* > *eef1a1l1* > *β2m* > *sdha* > *hmbsb* > *tubα1b* > *18S rRNA* > *gapdh*. As for the BestKeeper output, the rank was as follows: *18S rRNA* > *eef1a1l1* > *rpl13α* > *actβ2* > *polr2d* > *tbp* > *hmbsb* > *gapdh* > *β2m* > *sdha* > *tubα1b.* The M values of candidate reference genes in the 96 hpf embryo mitoxantrone test were generally higher than those in the 48 hpf embryo mitoxantrone test, suggesting that the expression levels were fluctuant. The most stable genes included *eef1a1l1*, *rpl13α*, and *tbp*. As the expression of *tbp* was classified as low transcript abundance, the present study suggests that *eef1a1l1* and *rpl13α* should be employed as reference genes for the 96 hpf zebrafish embryo mitoxantrone test.

### 2.3. Expression Normalization and Comparison of Target Genes Based on Gapdh Internal Control

**Figure 2:**
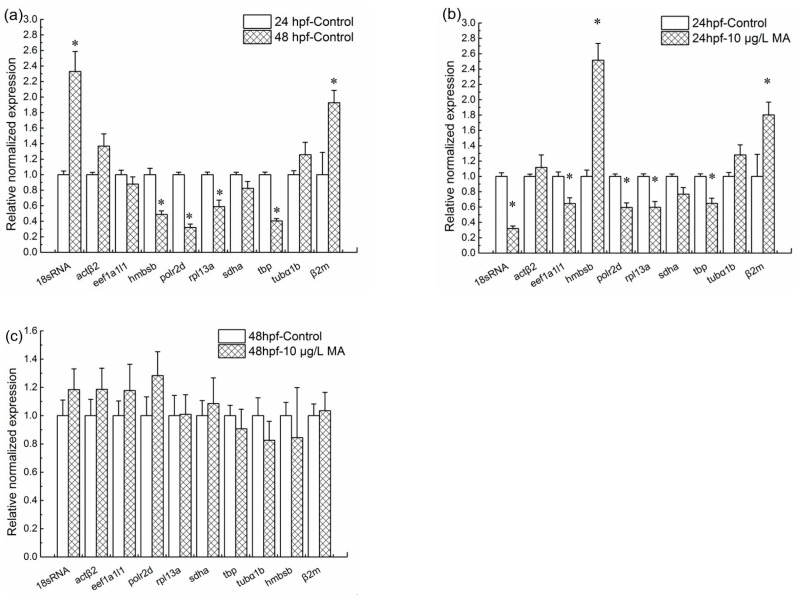
Normalization expression analysis of a set of target genes with *gapdh* as a reference gene. (**a**) The relative normalized expression levels of target genes at 48 hpf unexposed embryos comparing to 24 hpf unexposed embryos; (**b**) the relative normalized expression levels of target genes at 24 hpf embryos with 10 μg/L mitoxantrone treatment comparing to 24 hpf unexposed embryos; (**c**) the relative normalized expression levels of target genes at 48 hpf embryos with 10 μg/L mitoxantrone treatment comparing to 48 hpf unexposed embryos. Each group was conducted in triplicate (*n* = 3). All values are expressed as means ± standard error of the mean (SEM). * represents significant difference compared to control, *p* < 0.05.

The expression levels of a set of target genes were normalized with *gapdh*. The target set included the remaining ten genes—*18S rRNA*, *polr2d*, *tbp*, *hmbsb*, *rpl13α*, *actβ2*, *tubα1b*, *eef1a1l1*, *sdha*, and *β2m*—used in this study. To analyze the inducement factors for expression levels changing of target changes, the comparisons between embryos at 24 hpf and 48 hpf, exposed and unexposed embryos at 24 hpf, exposed and unexposed embryos at 48 hpf were conducted respectively. Compared to the 24 hpf control (0 μg/L mitoxantrone) groups, the normalized expression levels of four genes, including *hmbsb*, *polr2d*, *rpl13α* and *tbp* significantly decreased (*p* < 0.05), while two genes *18S rRNA*, *β2m* increased significantly (*p* < 0.05) at the 48 hpf control groups (Figure 2a); the normalized expression levels of five genes, including *18S rRNA*, *eef1a1l1*, *polr2d*, *rpl13α* and *tbp* decreased significantly, while *hmbsb* and *β2m* increased significantly at 24 hpf embryos with 10 μg/L mitoxantrone treatment (Figure 2b). Notably, compared to the 48 hpf control groups, the normalized expression levels of *18S rRNA*, *polr2d*, *tbp*, *hmbsb*, *rpl13α*, *actβ2*, *tubα1b*, *eef1a1l1*, *sdha*, and *β2m* showed no changes at 10 μg/L mitoxantrone exposed 48 hpf embryos (Figure 2c).

### 2.4. The Expression Analysis of Toxicity Biomarkers in Embryonic Zebrafish to Mitoxantrone Exposure

**Figure 3:**
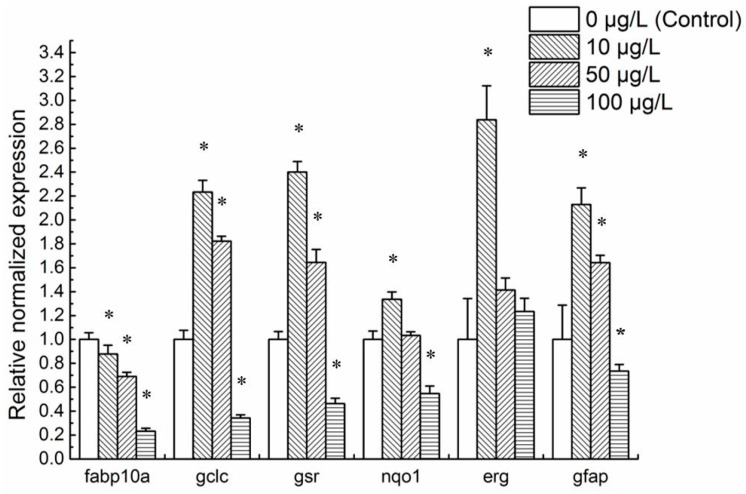
The expression analysis of classical toxicity biomarker genes in 48 hpf embryonic zebrafish exposed to mitoxantrone. Each group is conducted in triplicate (*n* = 3). All values are expressed as means ± standard error of the mean (SEM). * represents significant difference compared to the corresponding control (0 μg/L mitoxantrone) groups, *p* < 0.05.

The normalized expression levels of common zebrafish toxicity biomarker genes were analyzed in 48 hpf embryonic zebrafish to gradient concentrations of mitoxantrone (Figure 3). The maximum non-lethal concentration for 96 h (96 h-LC_0_) was 100 μg/L mitoxantrone. Therefore, we conducted present research on lower concentrations illuminating the sublethal effect of mitoxantrone on embryo development. The data showed that the expression of *fabp10a*, *gclc*, *gsr*, and *nqo1* changed significantly in 48 hpf embryos exposed to mitoxantrone. Compared to the control, the mRNA levels of *gclc*, and *gsr* of 48 hpf embryos to 10 and 50 μg/L mitoxantrone were higher, while their mRNA levels in 100 μg/L mitoxantrone group were lower. The mRNA levels of *fabp10a* decreased as exposure concentrations increased. These results suggest that mitoxantrone potentially induces hepatotoxicity in the developmental period of zebrafish embryos.

The classic neurotoxicity biomarker gene *gfap* was also influenced by mitoxantrone exposure. The mRNA levels of *gfap* increased significantly in 10 and 50 μg/L mitoxantrone-treated 48 hpf embryos and decreased in the 100 μg/L mitoxantrone group. These results suggest that mitoxantrone induces neurotoxicity in the embryonic development period of zebrafish. 

The mRNA levels of *erg* increased in 48 hpf embryos exposed to 10 μg/L mitoxantrone. As the mitoxantrone concentrations rose to 50 and 100 μg/L, the expression of *erg* became commensurate with the control. 

### 2.5. Liver Histopathology Analysis

**Figure 4:**
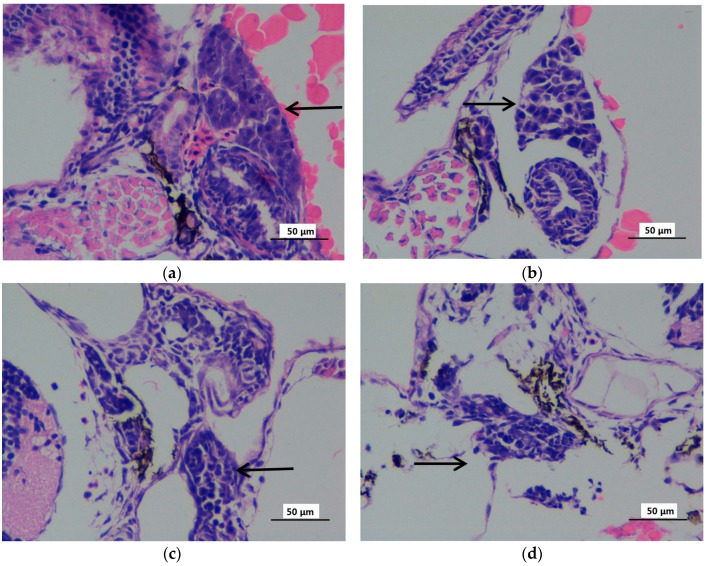
The hematoxylin-eosin staining microphotographs of zebrafish liver (72 hpf) exposed to gradient concentrations of mitoxantrone (×400). The zebrafish embryos exposed to 0 (**a**), 10 (**b**), 50 (**c**) and 100 (**d**) μg/L mitpxantrone were observed at 72 hpf with hematoxylin–eosin staining. The black arrow indicated the liver tissues. The scale bar indicated 50 μm.

The liver histopathology was analyzed at 72 hpf zebrafish embryos when the liver tissues histogenesis was completed. Liver hematoxylin-eosin (HE) staining showed abnormal pathological observation, including irregularly and loosely arranged liver tissue, vacuolization, and swelling hepatocytes in the liver tissue of some zebrafish embryos (72 hpf) exposed to 10 μg/L mitoxantrone (Figure 4b). More serious pathological abnormity was observed in liver tissues of embryos (72 hpf) exposed to 50 and 100 μg/L mitoxantrone (Figure 4c,d), including irregular arrangement, reduced size, hepatocyte constriction and pyknosis. The histopathology analysis confirmed the mitoxantrone-induced hepatotoxicity under gradient concentrations exposure, which was consistent with results from the expression analysis of the toxicity biomarkers.

### 2.6. Spontaneous Embryo Movement

**Figure 5:**
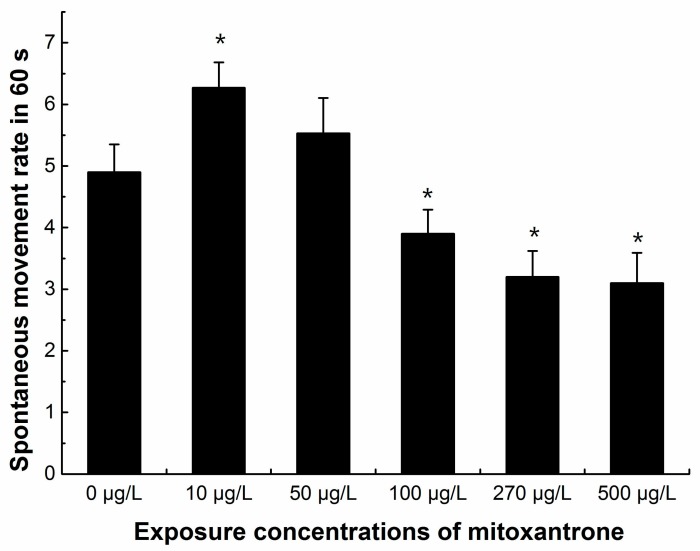
The numbers of spontaneous movement of zebrafish embryos (24 hpf) in 60 s. Eight embryos in each group were chosen for counted, and each group was conducted in six biological repeats (*n* = 48 totally in each group). All values are expressed as means ± standard error of the mean (SEM). * represents significant difference compared to control, *p* < 0.05.

Neurotoxicity was also observed with decreased spontaneous movement. The spontaneous movement was recorded in 24 hpf zebrafish embryos every 60 s. The data showed that the spontaneous movement rates increased significantly in 10 μg/L mitoxantrone exposed embryos (*p* < 0.05), and the spontaneous movement rates decreased significantly when zebrafish embryos were exposed to mitoxantrone concentrations higher than 100 μg/L in a dose-dependent manner (*p* < 0.05) (Figure 5).

## 3. Discussion

### 3.1. The Parameters Related with Reference Gene Stability

qPCR data analysis first involved subtracting the Cq values of the internal controls from the Cq values of target genes to obtain ΔCt. ΔCt values were then compared to the control group for ΔΔCt or further analysis. The Cq values of internal controls and target genes were the algorithm basis of the relative quantitative real-time PCR assay. The Cq value represented the initial concentration of the template. If the concentration became too high, the amplification would be insufficient; if the concentration was too low, the amplification curve would be non-linear. Among the 11 candidate genes in the study, *eef1a1la*, *rpl13α*, *actβ2*, *gapdh*, *polr2d*, and *tubα1b* were classified as median transcript abundance genes, which were more appropriate as reference genes. 

Normalization in relative quantitative qPCR assay was based on the linear relationship of Cq values between target genes and reference genes; namely, the regression coefficient was 1 [26]. The deviation of the regression coefficients from 1 resulted from the drift of E values (theoretically 100%) of reference genes and target genes. E values far from 100% might yield misleading results. In this study, we estimated E values of all primers using the dilution series method (calibration curve), confirming that E values ranged from 90 to 110%. Thus, the regression coefficient was nearly 1. 

Another important parameter was the correlation coefficient (R^2^). R^2^ represented the correction of reference gene abundance and the total amount of mRNA/cDNA presented in the samples. In this study, R^2^ values of all genes were more than 0.980, meaning a high level of positive linear relation between candidate reference gene abundance and the total amount of samples.

### 3.2. Validation of Reference Genes Based on Three Independent Algorithm Programs

In the present study, NormFinder [23], geNorm [24] and BestKeeper [25] were used respectively to grade and to rank the stability of given candidate reference genes. The independent algorithm programs suggested *gapdh* as the most stable reference gene, followed by *tubα1b*, for the 48 hpf zebrafish embryo mitoxantrone test. As for the 96 hpf zebrafish embryo mitoxantrone test, both *eef1a1l1* and *rpl13α* genes were suggested as reference genes with more stability than the other candidates. There were some differences in stability rank of candidate reference genes from three algorithm programs, which was acceptable because of their difference in the raw data input and mathematical approach.

The *gapdh* gene belonged to the earliest validated reference genes in zebrafish embryos, including cross-subfamily cloned embryos [27]. In the present study, the mRNA level of *gapdh* was stable in the 48 hpf zebrafish embryos exposed to mitoxantrone. In addition, its expression in zebrafish embryos exposed to Microcystin-LR [28] was constant. In various tissues of adult zebrafish, *gapdh* was also a recommendatory reference gene [22]. Recently, RNA-seq analysis showed that *gapdh* expression remained unchanged in adult zebrafish upon TDCIPP exposure [19]. According to present research, combined with these previous reports, it is highly speculated that the mRNA levels of zebrafish *gapdh* were relatively stable in the embryo stage.

In zebrafish, *eef1α1l1*, encoding eukaryotic translation elongation factor 1 alpha 1 for protein translation, was a relatively stable reference gene. The gene *eef1α1l1* has been reported as a reference gene for ovarian follicles of adult zebrafish [29], embryos [30], and larvae exposed to BPA structural analogs [31]. Similarly, BestKeeper and NormFinder in the present study suggested *eef1α1l1* as a reference gene for the 96 hpf zebrafish embryo mitoxantrone test. 

In early development research among various tissues of zebrafish embryos (<48 hpf), *rpl13α* was reported as a reference control in qPCR [32]. In more reports, *rpl13α* was used as a reference gene with other genes. In a locomotor activity assessment of 7 dpf zebrafish embryos, *rpl13α* was combined with *eef1α* as a reference gene for normalization [33]. In fast myotomal muscle fibers recruitment research of adult zebrafish, *rpl13α* and *actβ2* were employed for normalization in qPCR [21]. In research on adult zebrfish exposed to 17α-ethinylestradiol, *tubα1b* served as a reference gene along with *actβ2* and *eef1α* [34]. In practical use, no genes can act as a universal reference under different experimental conditions [16,17]. Usually, a combination of two or more stable reference genes as an internal control for normalizing is suggested. However, the combination of *gapdh* and *tubα1b* was not recommended, due to its less stability value than single *gapdh* gene in 48 hpf embryos mitoxantrone test.

The present research confirmed again that not all housekeeping genes were expressed constantly under different time courses, tissues, and experimental conditions. One of the most frequently used reference genes of zebrafish, *actβ2* [18,21,34,35,36,37,38], was found to be inappropriate as an internal control in the present study. The mRNA levels of *actβ2* varied between 24 and 48 hpf. Similarly, *18S rRNA*, *tbp*, *polr2d*, and *β2m* were unstable during the embryonic development period of zebrafish. The combination of *eef1a1l1* and *rpl13α* was advisable for the internal control in the 96 hpf zebrafish embryo mitoxantrone study.

### 3.3. Evaluation of Toxicity and Biomarker of Embryonic Zebrafish to Mitoxantrone

Representative toxicity biomarkers of liver included *gclc*, *gsr*, *nqo1*, and *fabp10a*. The proteins encoded by gene *gclc* (*glutamate-cysteine ligase, catalytic subunit*), *gsr* (*glutathione reductase*), and *nqo1* (*NAD(P)H dehydrogenase*, *quinone 1*) all participated in oxidation and detoxification in liver tissue, whose abnormal expression represented hepatotoxicity [39]. The catalytic subunit of glutamate-cysteine ligase, encoded by *gclc*, catalyzed the formation of gamma glutamate-cysteine from l-glutamate and l-cysteine. The expression and activity of GCLC reflected susceptibility to oxidative stress [40]. The *gsr* gene encoded for glutathione-disulfide reductase (also named glutathione reductase), an enzyme catalyzing the reduction of glutathione disulfide to the sulfhydryl form glutathione. The latter was a critical molecule in resisting oxidative stress [41]. The high levels of *gsr* expression suggested the increase levels of glutathione, indicating the activity of antioxidant defense system, protecting cells from damage from mitoxantrone. The gene *nqo1* encoded for a cytoplasmic 2-electron reductase DT-diaphorase, reducing quinones to hydroquinones [14]. The overexpression of NQO1 was reported to participate in the p53 stability regulation mechanism by increasing the content of NAD+, preventing the canceration of cells [42]. Also, NQO1 played a key role in ubiquinone and vitamin E quinone metabolism. These quinones protected cellular membranes from peroxidative injury in their reduced state [43]. The expression of *gclc*, *gsr*, and *nqo1* was induced by low concentrations of mitoxantrone other than high concentrations, implying different action modes of mitoxantrone in liver. The gene *fabp10a* (*fatty acid binding protein 10a*, *liver basic*), encoding fatty acid binding protein, was exclusively expressed in zebrafish liver [44]. The coding protein transported proteins for fatty acids and other lipophilic substances [45]. *fabp10a* was widely used as a hepatotoxicity biomarker in zebrafish [46,47]. Mitoxantrone exposure inhibited the expression of *fabp10a* mRNA in 48 hpf embryonic zebrafish in a dose-dependent manner. The data suggest that *fabp10a* might act as a biomarker of zebrafish embryos to mitoxantrone. The expression level of *fabp10a* decreased with the rise of mitoxantrone concentrations, suggesting mitoxantrone hepatotoxicity. Histopathological analysis of liver tissue showed hepatopathy, confirming the mitoxantrone-induced hepatotoxicity, even at a low exposure concentration. The results were consistent with previous rodent researches. Hepatotoxic signs, reduced hepatic levels and increased oxidized glutathione, and decreased ATP hepatic levels were reported in rat liver with mitoxantrone treatment, confirming the mitoxantrone-induced hepatotoxicity in rat [48]. 

According to previous cell experiments in vitro, mitoxantrone is oxidized by a cytochrome P450-mediated reaction generating quinone intermediates and quinonediimine metabolites [13]. ROS increase and mild oxidative stress were reported after mitoxantrone treatment in rat H9c2 cells [49]. In present study, with the increase expression levels of oxidation and detoxification related genes together with histopathological results, we inferred that mitoxantrone induced oxidative damage in zebrafish embryo liver in vivo.

The *erg* gene, also named the *ether-à-go-go-related* gene, was expressed in the early development stage of zebrafish embryos [50]. The *erg* gene coded for the alpha subunit of a potassium ion channel, contributing to the electrical activity of the heart [51]. *erg* was a popular toxicity biomarker for the heart [52]. Compared to the control, the expression level of the *erg* gene in embryonic zebrafish exposed to mitoxantrone showed no significant change. 

In this study, neurotoxicity was assessed according to the mRNA level of *gfap*. Glial fibrillary acidic protein (GFAP) is a type III intermediate filament protein exclusively expressed in astrocytes (AS) and considered a particular component of AS [53]. Thus, the expression of the *gfa*p gene was associated with nervous system development. The *gfa*p gene was widely adopted as a neurotoxicity biomarker in zebrafish [54]. The mRNA level of *gfap* in 48 hpf embryonic zebrafish was affected by mitoxantrone. These results suggest that mitoxantrone might induce neurotoxicity in embryonic zebrafish. Mitoxantrone exposure decreased the rate of spontaneous zebrafish embryo movement in the study, confirming that mitoxantrone had potential neurotoxic effects on zebrafish embryo development. Spontaneous embryo movement was a result of the combined action of muscle and neural systems. Spontaneous zebrafish embryo movement has been employed broadly as a toxicological endpoint for the chemical assessment on fish [55]. The rate of spontaneous zebrafish embryo movement decreased after exposure to herbicide cyhalofop-butyl [56], bactericide difenoconazole [57], and heart medications propranolol [58], but increased after pesticide biphenthrin exposure [59]. The underlying mechanism might be related to the protein channels on the cell membrane. For example, propranolol inhibited spontaneous movement by reducing the activity of sodium channels [59], whereas biphenthrin activated the spontaneous movement by elongating the open time of protein channels [60].

In conclusion, the most stable gene was *gapdh*, followed by *tuba1b*, in the 48 hpf embryo mitoxantrone test. Both *eef1a1l1* and *rpl13α* are recommended as reference genes in the 96 hpf zebrafish embryo mitoxantrone test. The single gene *gapdh* was proposed as a reference gene in the zebrafish embryo toxicity test of mitoxantrone after stability re-evaluation and comparison. With *gapdh* as an inner control, the mRNA levels of representative hepatotoxicity toxicity biomarkers, such as *fabp10a*, *gclc*, *gsr*, *nqo1*, and neurotoxicity biomarker *gfap* were changed after mitoxantrone exposure, indicating that mitoxantrone induced hepatotoxicity and neurotoxicity. Liver pathological analysis and spontaneous embryo movement supported these results. The transcript levels of *fabp10a* decreased in a dose-dependent manner, establishing a potential biomarker of mitoxantrone hepatotoxicity in zebrafish embryos. The data suggest that mitoxantrone might induce toxicity during the development of the liver and nervous system in embryonic zebrafish.

## 4. Materials and Methods

### 4.1. Zebrafish Maintenance

Wild type zebrafish AB (*Danio rerio*) parents were purchased from China Zebrafish Resource Center (CZRC) and maintained in our laboratory. The husbandry protocol was in accordance with the *Zebrafish Book* [61]. The zebrafish facility (ESEN EnvironScience, Beijing, China) was maintained under a day/night cycle of 14 h/10 h, 28 ± 0.5 °C with water conditions of 500–550 μS/cm and pH 7.0–7.5. Fish were fed live brine shrimp 3 times a day.

### 4.2. Zebrafish Embryo Toxicity Test

Mitoxantrone hydrochloride (CAS: 70476-82-3, M6545) was purchased from Sigma. Mitoxantrone was dissolved in an E3 buffer (5 mM NaCl, 0.17 mM KCl, 0.33 mM CaCl_2_, 0.33 mM MgSO_4_) to a final concentration of 1 g/L [62]. Stock solution was stored in the dark at 4 °C. Before the test, stock solution was diluted with an E3 buffer to working concentrations (0, 10, 50 and 100 μg/L) and pre-conditioned at 28 °C. The maximum non-lethal concentration for 96 h (96 h-LC0) was 100 μg/L mitoxantrone. The zebrafish embryo toxicity test was an improvement on the *fish embryo acute toxicity (FET) test*, based on OECD 236 guidelines [55]. Healthy wild-type zebrafish, at a 1:1 male/female ratio, were placed in segregated spawning tanks the day before the FET experiment. The tanks were made of food grade plastic and transparent. Each tank has a capacity of 3 L. Dividers were removed after the onset of the light cycle the next day, and embryos were collected 30 min afterward. Embryos were rinsed twice in the E3 buffer before the observation and selection of fertilized, synchronously developed eggs under stereomicroscopy Model S8 AP0 (Carl Zeiss, Oberkochen, Germany). Selected embryos were randomly transferred into pre-conditioned 6-well plates 703001 (Nest Biotechnology, Wuxi, China) at 3 hpf. The plates were put into 28 ± 0.5 °C chamber one hour ahead of exposure. Each well was distributed with 10 embryos and 6 mL of 0 (E3 buffer control), 10, 50 and 100 μg/L freshly diluted mitoxantrone. Each plate with a concentration of mitoxantrone or an E3 buffer control was considered a group. Each group was carried out in triplicate. All plates were conditioned at 28 ± 0.5 °C in a 14 h light/10 h dark chamber. 70% volume of solutions in each well were replaced, and dead embryos were abandoned every 24 h. 

### 4.3. RNA Extraction and Reverse Transcription

Embryos from the control groups and the mitoxantrone treatment groups were collected at 24, 48, 72, and 96 hpf, separately. Discarded the exposure solutions as much as possible. Thirty embryos in each 1.5 mL Eppendorf tube were immediately frozen in liquid nitrogen. RNA was extracted with TRizol reagent (Invitrogen, Shanghai, China) following the manufacturer’s instructions. RNA was dissolved in RNase-free water and qualified by Nanodrop 2000 and agarose gel electrophoresis. Qualified RNA was reversely transcripted for cDNA via PrimeScript RT reagent Kit with gDNA Eraser (TaKaRa, Dalian, China) following the manufacturer’s instructions. All samples were stored at −80 °C.

### 4.4. Primer Design and Quantitative Real-Time PCR

A set of candidate reference genes were chosen according to published articles for zebrafish embryo study (Table 4). These genes were reported as an internal control in qPCR studies of zebrafish under various conditions. The primer designing procedure is as follows: (a) Search the sequence information of reported genes or their orthologue genes in zebrafish. (b) Pick up primers based on the online software Primer3web version 4.0.0 (http://bioinfo.ut.ee/primer3/, accessed on 20 April 2016). (c) Verify primer specificity via the online software Primer-BLAST (http://www.ncbi.nlm.nih.gov/tools/primer-blast/index.cgi?LINK_LOC=BlastHome, accessed on 20 April 2016). (d) Synthesize primers with Shanghai Sangon.

All primer pairs were testified for amplification efficiency (E) and correlation coefficient (R^2^). Zebrafish cDNA of the 24 hpf control group was gradually diluted as 5^0^ (original gradient), 5^−1^, 5^−2^, 5^−3^, and 5^−4^. With the gradients and negative control as a template, all primers were testified in a CFX96 qPCR (Bio-Rad, Hercules, CA, USA) assay for an amplification curve. Based on the amplification curve, samples from both control and treatment groups (24, 48, 72, and 96 hpf) were diluted to a certain concentration and carried out for the qPCR assay. All reaction systems (25 μL) were as follows: 12.5 μL of SYBR Green Premix (Tiangen, Beijing, China), 2 μL of cDNA template, primer forward/reverse 0.5 μM, add MilliQ water up to 25 μL. The reaction procedure was as follows: 95 °C for 15 min; 95 °C for 10 s, and 60 °C for 30 s. 40 cycles. Followed a melt curve procedure: 65–95 °C with increments of 0.5 °C every 5 s. Quantification cycle (Cq) cutoffs of 40 were applied. All reactions were carried out in triplicates.

### 4.5. Stability Evaluation of Candidate Reference Geness

Amplification curves were analyzed for primer quality via CFX Manager software (Bio-Rad). Efficiency, R^2^, and Cq values and relative quantities were collected to evaluate the transcript abundance and variability. Three independent algorithm programs: geNorm [24], NormFinder [23], and BestKeeper [25] analyzed the stability of the reference gene set, separately.

### 4.6. Expression Normalization of Target Genes Based on Gapdh as Internal Control

The stability of the recommended most stable gene *gapdh* was re-evaluated and compared with Bio-Rad CFX Manager software. The expression of a set of zebrafish embryo target genes was normalized with the suggested reference gene *gapdh* as an internal control.

### 4.7. Expression Analysis of Toxicity Biomarkers in Embryonic Zebrafish Exposure to Mitoxantrone

The expression levels of classical toxicity biomarker genes *fabp10a*, *gclc*, *gsr*, *nqo1*, *gfap*, and *erg* in 48 hpf embryonic zebrafish exposed to gradient mitoxantrone (0, 10, 50, and 100 μg/L) were analyzed with *gapdh* as internal control. The primers (Table 5) were designed and synthesized as described in Section 2.4. All reaction systems (25 μL) were as follows: 12.5 μL of SYBR Green Premix (Tiangen, Beijing, China), 2 μL of cDNA template, and 0.5 μM primer forward/reverse. Up to 25 μLof MilliQ water was added. The reaction procedure was as follows: 95 °C for 15 min; 95 °C for 10 s, and 60 °C for 30 s. 35 cycles. 65–95 °C with increments of 0.5 °C every 5 s for melt curve. Quantification cycle (Cq) cutoffs of 35 were applied. All reactions were carried out in triplicates.

### 4.8. Liver HE Staining

During the toxicity test, three exposed embryos in each group (0, 10, 50 and 100 μg/L) at were collected for HE staining at 72 hpf. At 72 hpf, when the liver tissues developed and functioned completely, zebrafish embryos were collected without exposure solutions and infiltrated in 4% paraformaldehyde for tissue fixation in the dark for 24 h. The tissue biopsies and staining protocol were conducted on the basis of the previous reports [68]. After dehydration with gradient ethanol and dimethylbenzene, tissues were immersed in liquid paraffin at 65 °C followed by cooling at −20 °C. Embedded tissues were trimmed, sliced (to a thickness of 3 μm), and stored at room temperature. The tissue slides were rehydrated with dimethylbenzene and gradient ethanol for hematoxylin (Sinopharm CAS#17372-87-1, Beijing, China) and eosin (Sinopharm CAS#517-28-2, Beijing, China) staining. Finally, slides were mounted in balsam neutral for storage and microscopic observation (Olympus BX53, Tokyo, Japan).

### 4.9. Microscopic Observation and Counting

The zebrafish embryo toxicity test was conducted from 3 hpf in pre-conditioned 6-well plates. Each well was distributed with 10 embryos and 6 mL of 0 (E3 buffer control), 10, 50, 100, 270 and 500 μg/L freshly diluted mitoxantrone. Each plate with a concentration gradient or an E3 buffer control was considered a group. All plates were conditioned at 28 ± 0.5 °C in a 14 h light/10 h dark chamber. Each group was repeated in six biological repeats. Eight embryos in each group (0, 10, 50, 100, 270, and 500 ug/L mitoxantrone) were observed under microscope at 24 hpf. The rates of embryo (*n* = 48 totally in each group) spontaneous movement during 60 s were recorded. All values were expressed as means ± standard error of the mean (SEM).

### 4.10. Statistical Analysis

A statistical analysis and drawing was conducted, mainly based on OriginPro 8.0. The data was analyzed by the Levene test and two-way analysis of variance (ANOVA) at a significant level of 5% (*p* < 0.05). A post-hoc test after the ANOVA was conducted in SPSS 17.0. Multiple comparisons between the groups were performed using the Student–Newman–Keuls test method.

## Figures and Tables

**Table 1:** Transcript abundance and amplification efficiency of candidate reference genes.

Gene Name	E	R^2^	Mean Cq Value	Transcript Abundance
*18S rRNA*	0.941	0.999	9.44	High transcript abundance
*eef1a1l1*	0.946	0.999	19.46	Medium transcript abundance
*rpl13α*	1.016	0.998	20.53	
*actβ2*	0.957	0.999	21.58	
*gapdh*	1.016	0.993	22.69	
*polr2d*	0.922	0.994	25.4	
*tubα1b*	1.001	0.993	25.86	
*sdha*	0.963	0.997	27.92	Low transcript abundance
*tbp*	0.972	0.996	27.97	
*hmbsb*	0.901	0.987	29.56	
*β2m*	0.959	0.984	31.7	

**Table 2:** Stability analysis of candidate reference genes in the zebrafish embryo mitoxantrone test before 48 hpf *.

M Value *	NormFinder	geNorm	BestKeeper
*rpl13α*	0.021	0.678	1.70
*actb2*	0.013	0.668	1.22
*polr2d*	0.020	0.842	2.00
*18sRNA*	0.034	1.517	0.37
*hmbsb*	0.026	1.266	2.37
*sdha*	0.012	0.589	1.48
*tbp*	0.012	0.814	2.00
*β2m*	0.029	0.790	1.31
*eef1a1l1*	0.009	0.667	1.35
*gapdh*	0.007	0.574	1.45
*tubα1b*	0.010	0.613	1.46

* The higher the M value, the less stable the gene. The three most stable genes were marked with the underline for each algorithm.

**Table 3:** Stability analysis of candidate reference genes in the zebrafish embryo mitoxantrone test before 96 hpf *.

M Value *	NormFinder	geNorm	BestKeeper
*gapdh*	0.083	3.910	1.93
*sdha*	0.008	1.731	2.05
*eef1a1l1*	0.002	1.578	1.17
*rpl13α*	0.004	1.478	1.43
*actβ2*	0.004	1.529	1.56
*tubα1b*	0.034	2.139	2.62
*tbp*	0.001	1.514	1.61
*polr2d*	0.002	1.539	1.56
*β2m*	0.005	1.666	1.93
*hmbsb*	0.009	1.761	1.74
*18S rRNA*	0.080	2.948	0.74

* The higher the M value, the less stable the gene. The three most stable genes were marked with the underline for each algorithm.

**Table 4:** Candidate reference genes of zebrafish embryos used in the toxicity test.

Gene Symbol	Gene Name	Accession	Function	Primer Sequence (5′–3′)
*18S rRNA* [63,64]	*18S ribosomal RNA*	Generic	18S ribosomal RNA	F: cacttgtccctctaagaagttgca R: ggttgattccgataacgaacga
*eef1a1l1* [29,30,31]	*eukaryotic translation elongation factor 1 alpha 1, like 1*	NM_131263.1	Factor for protein translation	F: CTGGAGGCCAGCTCAAACAT R: ATCAAGAAGAGTAGTACCGCTAGCATTAC
*rpl13α* [58,61]	*ribosomal protein L13a*	NM_212784	Genetic Information Processing	F: TCTGGAGGACTGTAAGAGGTATGC R: AGACGCACAATCTTGAGAGCAG
*actβ2* [18]	*actin, beta 2*	NM_181601.4	Cytoskeletal structural protein	F: TCTGGTGATGGTGTGACCCA R: GGTGAAGCTGTAGCCACGCT
*Gapdh* [19,27]	*glyceraldehyde-3-phosphate dehydrogenase*	NM_001115114.1	Catalytic enzyme in glycolytic pathway	F: GATACACGGAGCACCAGGTT R: CAGGTCACATACACGGTTGC
*polr2d* [27]	*polymerase (RNA) II (DNA directed) polypeptide D*	NM_001002317.2	Enzyme for transcription	F: CCAGATTCAGCCGCTTCAAG R: CAAACTGGGAATGAGGGCTT
*tubα1b* [65]	*tubulin, alpha 1b*	NM_194388	Cytoskeletal structural protein	F: TGGAGCCCACTGTCATTGATG R: CAGACAGTTTGCGAACCCTATCT
*Sdha* [22]	*succinate dehydrogenase complex, subunit A, flavoprotein (Fp)*	NM_200910	Enzyme in tricarboxylic acid cycle	F: GAGTCTCCAATCAGTATCCAGTAGTAGA R: CACTGTGTGCGAGCGTGTTG
*Tbp* [66]	*TATA box binding protein*	NM_200096.1	Transcription factor	F: CTTACCCACCAGCAGTTTAGCAG R: CCTTGGCACCTGTGAGTACGACTTTG
*hmbsb* [22]	*hydroxymethylbilane synthase, b*	NM_001024388.1	Enzyme in heme synthesis	F: AAGAGCGTAATAGGCACCAGTTC R: GTTCTCCCAGCCCATTCTCTTC
*β2m* [67]	*beta-2-microglobulin*	NM_131163	Beta chain of a major histocompatibility complex I molecular	F: AGGATTGTCTGCTTGGCTCTCT R: GGAGTGGAGACTTTCCCCTGTAC

**Table 5:** The primers sequence of toxicity genes in embryonic zebrafish.

Gene Symbol	Gene Name	Accession	Primer Sequence (5′–3′)
*fabp10a*	*fatty acid binding protein 10a, liver basic*	NM_152960.1	F: CCAGTGACAGAAATCCAGCA R: GTTCTGCAGACCAGCTTTCC
*gclc*	*glutamate-cysteine ligase, catalytic subunit*	NM_199277.2	F: AAAATGTCCGGAACTGATCG R: AACGTTTCCATTTTCGTTGC
*gsr*	*glutathione reductase*	NM_001020554.1	F: CAACCTTGAAAAGGGCAAAA R: AAACTGGATCCTGGCACATC
*nqo1*	*NAD(P)H dehydrogenase, quinone 1*	BC065622.1	F: CTCAAGGATTTGCCTTCAGC R: CGCAGCACTCCATTCTGTAA
*gfap*	*glial fibrillary acidic protein*	NM_131373.2	F: CCTGACCTGTGACCTGGAAT R: TCCAGCAGCTTCCTGTAGGT
*erg*	*potassium voltage-gated channel, subfamily H (eag-related), member 6a*	NM_212837.1	F: CAGATGCTCCGTGTGAAAGA R: TGCGGTTCAGATGAAGACAG

## Abbreviations

CZRC	China Zebrafish Resource Center
OECD	Organization for Economic Co-operation and Development
qPCR	Quantitative Real-time PCR
